# Spatio-temporal temperature variations in MarkSim multimodel data and their impact on voltinism of fruit fly, *Bactrocera* species on mango

**DOI:** 10.1038/s41598-019-45801-z

**Published:** 2019-07-04

**Authors:** Jaipal Singh Choudhary, Santosh S. Mali, Debu Mukherjee, Anjali Kumari, L. Moanaro, M. Srinivasa Rao, Bikash Das, A. K. Singh, B. P. Bhatt

**Affiliations:** 1 ICAR-Research Complex for Eastern Region, Research Centre, Plandu, Ranchi, 834010 Jharkhand India; 2 ICAR- Central Research Institute for Dryland Agriculture (CRIDA) Santoshnagar, Saidabad PO, Hyderabad, 500 059 India; 3 ICAR-Research Complex for Eastern Region, ICAR Parisar, P. O. Bihar Veterinary College, Patna, 800 014 Bihar India

**Keywords:** Environmental health, Projection and prediction

## Abstract

Fruit flies are the most serious economic insect pests of mango in India and other parts of the world. Under future climate change, shifts in temperature will be a key driver of ecosystem function especially in terms of insect pest dynamics. In this study, we predicted the voltinism of the three economically important fruit fly species viz., *Bactrocera dorsalis* (Hendel), *Bactrocera correcta* (Bezzi) and *Bactrocera zonata* (Saunders) of mango from 10 geographical locations in India using well established degree day approaches. Daily minimum and maximum temperature data were generated by using seven General Circulation Models (GCMs) along with their ensemble, in conjunction with the four representative concentration pathways (RCPs) scenarios (RCP 2.6, RCP 4.5, RCP 6.0 and RCP 8.5) and three time periods (2020, 2050 and 2080) generated from MarkSim® DSSAT weather file generator. Historical data from 1969–2005 of these 10 locations were considered as baseline period. Under future predicted climates, model outputs indicates that all three fruit fly species will produce higher number of generations (1–2 additional generations) with 15–24% reduced generation time over the baseline period. The increased voltinism of fruit fly species due to increased temperature may lead to ≃5% higher infestation of mango fruits in India by the year 2050. Analysis of variance revealed that ‘geographical locations’ explained 77% of the total variation in voltinism followed by ‘time periods’ (11%). Such increase in the voltinism of fruit flies and the consequent increases in the infestation of mango fruits are likely to have significant negative impacts on mango protection and production.

## Introduction

Changing scenarios of climate is expected to have acute consequences for agriculture worldwide through its impacts on population dynamics, rate of invasion, suitability, extinction etc. of arthropods in natural and agricultural systems^1–3^. Fifth Assessment Report (AR5) of the Intergovernmental Panel on Climate Change (IPCC) through the Coupled Model Intercomparison Project Phase 5 (CMIP5) predicted significant future climate change and variability which would cause harsh impacts on various ecosystems. Changes in the temperature would be one of the most powerful drivers of the functionality of ecosystem caused by climate change^4^. The earth’s global average surface temperature increased by 0.78 °C over the 20^th^ century and expected up to 1.8–4 °C increase by 2100^5^. However, the amount of increase is expected to be different across geographical locations^6^. Global warming are likely to suffer agriculture more in low latitude and developing countries as compared to high latitude regions where climate change will have beneficial impact on agriculture^6^. Insect along with other arthropod individuals are designated as poikilothermic organisms that developmental rate is extremely dependent on external temperature conditions. The findings of various studies have already been documented that the development of insect pest would be very sensitive to temperature changes^6–8^ and even a small spatio-temporal change in temperature could influence the growth, survival, distribution, behavior and reproduction of insect pests^9–11^. The world is likely to witness major insect pest outbreaks due to round the year favourable climatic conditions and food availability for their multiplication. Therefore, it is important to assess the effects of global warming on insect pests and further their influence on changed dynamics on yield and yield loss of food crops. This will ensure future agricultural achievements through designing of most economic pest management strategies and to allow agricultural systems to be resilient to the changing climatic conditions.

Future predictions of insect pests on model based studies on region basis are very valuable methods for better preparedness to contest/counter the outbreaks of serious insect pests in changing climate scenarios^12,13^. Global circulation models (GCMs) are very sophisticated way of representing the climate projections in modelling studies^14^. Projections derived from GCMs have been used by several researchers to predict the voltinism (i.e., number of generations) using Growing Degree Day (GDD) approaches in forestry insect pests^15,16^ and agriculture pests^13^. For particular insect, the number of generations produced is the result of accumulation of required GDD, which is the amount of heat unit accumulated above a specified base temperature during a 24 hours period^17^. Insects are sensitive to temperature, such that a small increase in temperature results in severe impact on their annual number of generations^18^. In context of insect sensitivity to temperature, the simulated variations of temperature for the projected periods across 10 locations of the present study would be able to predict the effect on the insect development. The advantage of using ensemble output either with GCM model or with Representative Concentration Pathway (RCP)/emission scenarios is well known and is to eliminate the uncertainties associated with climate change projections^19^. The detailed information about these Representative Concentration Pathways (RCPs) can be found in van Vuuren *et al*.^20^. MarkSim is a web based stochastic climate simulation platform that generates daily and downscaling weather data of future time periods of different GCMs and scenarios^21^. MarkSim generated data can be used to handle any agricultural model which requires daily weather data.

Mango (*Mangifera indica* L.) is one of the preferred fruits having the characteristics of fabulous and delicious taste, flavor and sweet fragrance. India is the largest mango producing country in the world accounting ≃ 41% percent of the world’s mango production. The total annual revenue from mango export to other countries in India was about to Rs. 209 crores in 2010–11 (http://agriexchange.apeda.gov.in/Market%20Profile/one/MANGO.aspx). The tephritid fruit flies (Diptera: Tephritidae), are the most serious economic insect pests of mango around the world^22^ including India^23^. In India, it is estimated that fruit flies are causing 5–80% yield losses in mango directly or indirectly^24^. Nearly 48 species of fruit flies have been reported to attack/ infest on mango. Among them, *Bactrocera dorsalis* (Hendel), *Bactrocera correcta* (Bezzi) and *Bactrocera zonata* (Saunders) are the most widespread fruit fly species found infesting mango in India^25^.

Keeping in view the importance of the mango and fruit fly associated yield losses, we employed GDD approach with temperature data from MarkSim to predict the spatial and temporal changes in voltinism of fruit fly species (*B. dorsalis*, *B. correcta* and *B. zonata*) during future climate change periods on mango across different regions of India under various emission scenarios.

## Data and Methodology

### Sources of temperature data

The future projected time series data of daily minimum (MinT) and maximum temperature (MaxT) at ten study locations was downloaded from MarkSim® DSSAT weather file generator (http://gisweb.ciat.cgiar.org/MarkSimGCM/) for each combination of the seven GCMs and their ensemble under the four greenhouse gas concentration trajectories scenarios RCP 2.6, RCP 4.5, RCP 6.0 and RCP 8.5 with 20 replicates of each. The seven GCMs used in present study were the Chinese Beijing Climate Center, China Meteorological Administration and Analysis model BCC-CSM1-1(BC); the Australian Commonwealth Scientific and Industrial Research Organization model CSIRO-Mk3-6-0(CS); the Chinese the First Institute of Oceanography model FIO-ESM (FI); the US National Oceanic and Atmospheric Administration’s Geophysical Fluid Dynamics Laboratory model GFDL-ESM2M (GF); the UK’s Met Office Hadley Centre’s model HadGEM2-ES (Had); the French Institut Pierre-Simon Laplace model IPSL-CM5A-MR (IP); the Japan’s Atmosphere and Ocean Research Institute, National Institute for Environmental Studies and Agency for Marine-Earth Science and Technology model MIROC-ESM-CHEM (MI) and ensemble of above all and thus in total eight models were used. Further detailed information on MarkSim weather file generator can be found in Jones and Thornton^21^ and Jones *et al*.^26^. Historical daily temperatures data (baseline) for all the locations (Table 1) were collected from the India Meteorological Department (IMD) grid temperature data available at 1 × 1 degree resolution. It is understood that temperature forecast mainly depends on the type of models, scenarios and locations considered. Climate projections were studied over three time periods, viz., 2020 (considered for near future), 2050 (considered for distant future) and 2080 (considered for very distant future) and compared over Baseline (BL) (1969–2005) periods from each GCM model for ten locations. The projected temperature data of MaxT and MinT were collected for 3 climate change periods (2020, 2050 and 2080), 4 (RCP 2.6, RCP 4.5, RCP 6.0 and RCP 8.5) scenarios, across 8 models and at 10 mango growing locations of India to estimate generation time (i.e. degree days per generation) and voltinism for 3 fruit fly species.

**Table 1:** Variation of Annual mean (maximum and minimum) temperature among four representative concentration pathway scenarios across ten mango growing locations.

Scenario/variable	Time period	Lucknow	Mohanpur	Paria	Ranchi	Rewa	Rupnagar	Bengaluru	Vengurle	Sangareddy	Dharampuri
**Temperature (Maximum)**											
Baseline	(1969–2005)	31.89	31.05	33.13	31.03	32.22	28.76	30.26	31.14	33.09	32.25
RCP 2.6	2020	32.72 ± 1.28	31.69 ± 0.80	32.60 ± 0.69	29.80 ± 1.13	32.29 ± 1.31	31.87 ± 1.45	29.73 ± 0.69	31.74 ± 0.63	32.74 ± 0.94	32.64 ± 0.66
	2050	33.24 ± 1.27	32.09 ± 0.78	34.46 ± 0.71	30.25 ± 1.12	32.74 ± 1.19	32.39 ± 1.44	30.12 ± 0.67	32.15 ± 0.64	33.26 ± 0.94	33.03 ± 0.66
	2080	33.47 ± 1.29	32.28 ± 0.77	33.13 ± 0.68	30.46 ± 1.10	32.91 ± 1.26	33.92 ± 1.42	30.27 ± 0.67	32.26 ± 0.62	33.26 ± 0.93	33.05 ± 0.71
RCP 4.5	2020	32.80 ± 1.25	31.80 ± 0.82	32.62 ± 0.71	29.80 ± 1.13	32.34 ± 1.27	31.97 ± 1.49	29.78 ± 0.65	31.73 ± 0.66	32.67 ± 0.91	32.77 ± 0.67
	2050	33.77 ± 1.27	32.66 ± 0.75	34.58 ± 0.71	30.98 ± 1.14	33.28 ± 1.25	32.99 ± 1.47	30.54 ± 0.68	32.58 ± 0.69	33.63 ± 0.92	33.40 ± 0.64
	2080	34.61 ± 1.28	33.24 ± 0.71	34.03 ± 0.65	31.74 ± 1.12	33.99 ± 1.26	33.89 ± 1.46	31.34 ± 0.73	33.17 ± 0.64	34.43 ± 0.94	33.97 ± 0.72
RCP 6.0	2020	32.75 ± 1.27	31.70 ± 0.80	32.63 ± 0.70	29.74 ± 1.12	32.36 ± 1.28	31.90 ± 1.46	29.73 ± 0.70	31.74 ± 0.64	32.76 ± 0.94	32.70 ± 0.70
	2050	33.45 ± 1.28	32.24 ± 0.78	34.74 ± 0.72	30.53 ± 1.13	33.01 ± 1.29	32.60 ± 1.46	30.32 ± 0.65	32.36 ± 0.59	33.41 ± 0.91	33.22 ± 0.62
	2080	34.55 ± 1.27	33.11 ± 0.76	33.94 ± 0.65	30.97 ± 1.13	33.90 ± 1.29	33.88 ± 1.46	30.98 ± 0.66	33.05 ± 0.60	34.03 ± 0.91	33.82 ± 0.70
RCP 8.5	2020	32.82 ± 1.27	31.74 ± 0.81	32.52 ± 0.70	29.74 ± 1.16	32.33 ± 1.27	32.07 ± 1.49	29.74 ± 0.66	31.80 ± 0.65	32.73 ± 0.98	32.71 ± 0.66
	2050	33.45 ± 1.28	32.90 ± 0.78	35.24 ± 0.72	31.17 ± 1.12	33.62 ± 1.28	33.54 ± 1.44	30.87 ± 0.65	32.94 ± 0.60	34.05 ± 0.94	33.78 ± 0.63
	2080	36.18 ± 1.26	34.48 ± 0.76	35.41 ± 0.65	33.00 ± 1.09	35.55 ± 1.27	35.92 ± 1.43	32.54 ± 0.66	34.55 ± 0.62	35.85 ± 0.95	35.69 ± 0.72
**Temperature (Minimum)**											
Baseline	(1969–2005)	18.92	22.35	20.94	19.14	18.87	15.70	19.28	21.89	21.27	21.22
RCP 2.6	2020	20.25 ± 1.59	22.90 ± 1.10	22.68 ± 0.94	18.69 ± 1.26	19.82 ± 1.60	18.63 ± 1.68	18.74 ± 0.59	23.48 ± 0.66	21.78 ± 0.87	22.08 ± 0.57
	2050	20.75 ± 1.56	23.35 ± 1.10	22.84 ± 1.01	19.37 ± 1.22	20.26 ± 1.56	19.14 ± 1.66	19.33 ± 0.57	24.00 ± 0.66	22.32 ± 0.86	22.56 ± 0.57
	2080	20.90 ± 1.58	23.50 ± 1.08	23.35 ± 0.68	19.43 ± 1.10	20.35 ± 1.57	20.61 ± 1.62	19.44 ± 0.56	24.11 ± 0.64	22.46 ± 0.86	22.47 ± 0.62
RCP 4.5	2020	20.22 ± 1.55	22.99 ± 1.11	22.64 ± 0.94	18.70 ± 1.26	19.80 ± 1.57	18.72 ± 1.70	18.85 ± 0.55	23.49 ± 0.67	21.80 ± 0.85	22.14 ± 0.57
	2050	21.19 ± 1.56	23.80 ± 1.07	23.16 ± 1.00	19.91 ± 1.23	20.76 ± 1.55	19.76 ± 1.67	19.82 ± 0.56	24.30 ± 0.67	22.76 ± 0.84	22.94 ± 0.55
	2080	21.89 ± 1.54	24.36 ± 1.05	24.29 ± 0.86	20.53 ± 1.17	21.39 ± 1.53	20.62 ± 1.65	20.44 ± 0.57	24.96 ± 0.63	23.55 ± 0.82	23.52 ± 0.61
RCP 6.0	2020	20.15 ± 1.57	22.92 ± 1.10	22.61 ± 0.94	18.68 ± 1.24	19.76 ± 1.58	18.63 ± 1.68	18.81 ± 0.60	23.46 ± 0.66	21.79 ± 0.85	22.13 ± 0.60
	2050	20.97 ± 1.56	23.53 ± 1.08	23.03 ± 1.02	19.62 ± 1.21	20.46 ± 1.57	19.36 ± 1.67	19.55 ± 0.55	24.20 ± 0.63	22.57 ± 0.84	22.77 ± 0.54
	2080	22.08 ± 1.53	24.41 ± 1.09	24.51 ± 0.85	20.11 ± 1.21	21.69 ± 1.53	20.75 ± 1.66	20.58 ± 0.56	25.25 ± 0.62	23.61 ± 0.82	23.65 ± 0.63
RCP 8.5	2020	20.24 ± 1.57	22.98 ± 1.11	22.65 ± 0.93	18.61 ± 1.27	19.80 ± 1.57	18.78 ± 1.71	18.90 ± 0.56	23.30 ± 0.66	21.87 ± 0.88	22.19 ± 0.57
	2050	20.97 ± 1.56	24.21 ± 1.08	23.70 ± 0.99	20.42 ± 1.20	21.29 ± 1.54	20.33 ± 1.65	20.19 ± 0.54	24.74 ± 0.63	23.25 ± 0.84	23.40 ± 0.53
	2080	23.76 ± 1.48	25.79 ± 1.11	26.06 ± 0.81	22.42 ± 1.13	23.51 ± 1.46	22.74 ± 1.60	22.09 ± 0.53	26.48 ± 0.60	25.26 ± 0.80	25.14 ± 0.65

### Pest degree-day approaches and voltinism

**Figure 1:**
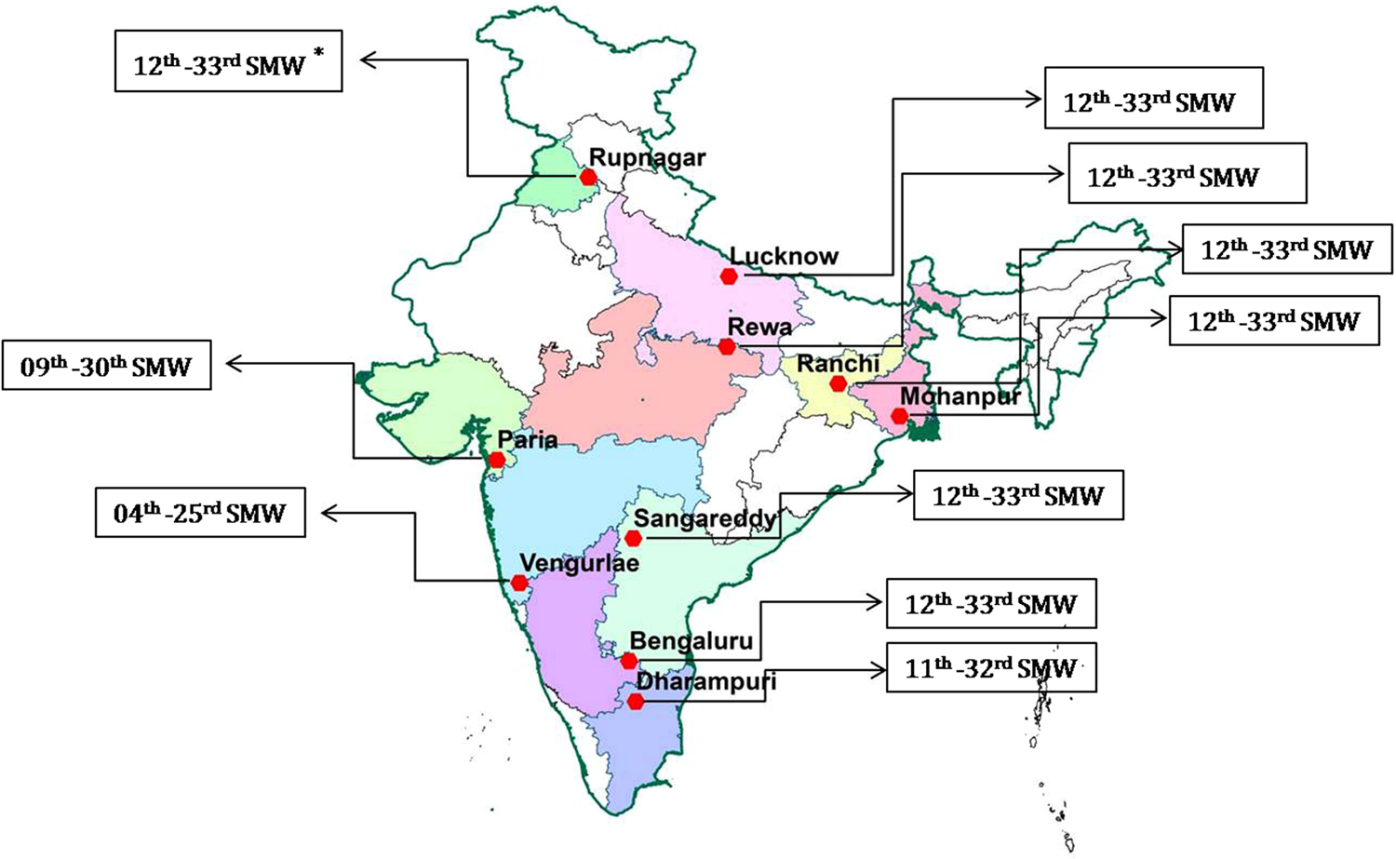
Major mango growing locations of India selected for prediction of number of generations of *Bactrocera* sp. during future climate change scenarios. The map was created in ArcGIS 10.2 software (ESRI Inc.). *Period (Standard Meteorological Weeks, SMW) considered for prediction of voltinism and generation time of *Bactrocera* sp.

We used Growing Degree Day (GDD) approach to predict the voltinism of three fruit fly species under future climate scenarios. The software ‘*ingen’* (Insect Generations) developed by Srinivasa Rao *et al*.^27^ and freely available at www.nicra.in was used to calculate the GDD. The software is employed with a default horizontal cut-off setting for upper developmental threshold and output is given in form of number of insect generations, number of degree days, insect generations with generation time during whole year, crop season or any particular period of the year. The lower developmental threshold and number of degree days for completing life cycle of *B. dorsalis*, *B. correcta* and *B. zonata* were 11.8 °C; 358 DD, 15.7 °C; 726 DD and 12.6 °C; 380 DD, respectively^28–30^. The duration of mango fruiting period from marble stage to harvesting time (fruit fly susceptible stages) usually takes 130 days in India. Considering this fact, the daily temperature data during the fruit fly susceptible stage of duration of 130 days with respective Standard Weeks of each location (Fig. 1) were considered for predicting the variation in generations and generation time of major mango fruit fly species.

Contribution of variation due to location, scenario, model, time period and their interactions in predicted number of generations was assessed using the variance partitioning by analysis of variance (ANOVA). The sum of squares of each term was divided by the total sum of squares elucidated by the model to obtain the individual contribution of each term. We did not make any statistical inferences from ANOVA because models used in present study are deterministic in nature^13^. The data on number of generations were analysed through one way ANOVA and means were compared using Tukey’s honestly significant difference (HSD) tests for comparison at probability level of 5%. All statistical analyses were performed using SPSS 21.0.

### Estimation of fruit fly infestation

The reports on estimated infestation in mango fruits due to fruit flies infestation under present and past conditions were compiled and normalized for locations studied. The literature reports were collected for each location. The reviewed reports for fruit infestation assessment are as follows: for Ranchi reports by Singh *et al*.^31^, Choudhary *et al*.^23^, for Bengaluru by Tandon and Verghese^32^ Veghese *et al*.^33^, Shukla *et al*.^34^ for Lucknow by Shukla *et al*.^34^, IMFFI Semi-Structured Interview Survey Report^35^, for Paria by Patel *et al*.^36^, Nayaka *et al*.^37^, IMFFI Semi-Structured Interview Survey Report^35^, Kumar *et al*.^38^, for Rupnagar by Mann^39^, Rewa by Dwivedi and Singh^40^, for Sangareddy by Kannon and Rao^41^, Mohanpur by Laskar *et al*.^42^, for Vengurle and Dharampuri (personal communication with experts at these locations). Based on the data and information available in these reports, a relationship was established using linear regression equation between estimated generation numbers and estimated average fruit infestation of mango under current climatic conditions in India^43^.

1 $$\hat{{\rm{Y}}}=\alpha +\beta X$$

Where $\hat{{\rm{Y}}}$ is the estimated fruit infestation (%) under current climatic conditions, $X$ is the corresponding number of generations, representing possible infestation due to fruit flies, and *α* and *β* are the intercept and slope of the regression line, respectively.

Further, the *ingen* was applied to estimate the number of generations of the fruit fly at all the study locations for the baseline period. Using the values of *α*, *β* and the estimated number of generations, the fruit infestation of mango at the study locations was estimated for the baseline and future climate change periods. Here, the assumption was that the relationship between number of generations and fruit infestation will remain valid and applicable for the future climatic conditions. Thus, the values for $X$ in the above equation were replaced with number of generations for the 2020, 2050 and 2080 periods as defined below:

2 $${\hat{{\rm{Y}}}}_{1,2,3}=\alpha +\beta {X}_{1,2,3}$$

where ${\hat{{\rm{Y}}}}_{1,2,3}$ is estimated fruit infestation (%) under future climatic conditions of the 2020 (1), 2050 (2) and 2080 (3), respectively, ${X}_{1,2,3}$ is corresponding number of generations representing fruit flies infestation potential under future climate change periods.

## Results

### Variation in projected temperature

Significant variation of future temperature (MaxT and MinT) projected by eight models including ensemble were observed when compared over four RCPs, three time periods and ten locations of the India presented in Tables 1 and S1. It is expected that MaxT would fluctuate by ±0.47 to ±4.02 °C and minimum temperature by ±0.43 to ±6.78 °C during three future climate change periods over Baseline period of four scenarios (RCP 2.6, RCP 4.5, RCP 6.0 and RCP 8.5) at ten locations of India (Table 1). The maximum increase in both maximum and minimum temperature was projected at Rupnagar followed by Lucknow in the northern part of India (Table 1). Highest temporal changes in temperature from 2020 to 2080 are predicted for Had model (Table S1) irrespective of scenarios.

### Variation in voltinism of *Bactrocera* sp. across locations, models and scenarios

Under each climate change scenario (RCPs), the projected number of generations of fruit flies across all the locations showed significant differences (Tables 2–4) in future climate periods over the baseline.

**Table 2:** Variation in number of generations (±standard deviation) of *Bactrocera dorsalis* on mango in four scenarios under future climate change periods.

Scenario/time period	Ranchi	Lucknow	Paria	Bengaluru	Vengurle	Sangareddy	Rewa	Rupnagar	Dharampuri	Mohanpur
Baseline	6.92^b^	8.28^b^	6.83^a^	6.03^d,e^	6.24^a^	7.36^a^	7.40^a^	6.30^a^	6.82^a^	7.00^a^
RCP 2.6/2020	6.67 ± 0.08^a^	8.01 ± 0.13^a^	7.11 ± 0.11^b^	5.61 ± 0.11^a^	6.49 ± 0.08^b^	7.44 ± 0.07^a,b^	7.89 ± 0.12^b^	7.72 ± 0.07^b,c^	6.96 ± 0.09^b^	7.38 ± 0.05^b^
RCP 2.6/2050	6.92 ± 0.11^b^	8.23 ± 0.14^b^	7.64 ± 0.12^d^	5.79 ± 0.13^c^	6.70 ± 0.11^c^	7.67 ± 0.14^b^	8.08 ± 0.15^c^	7.90 ± 0.14^c^	7.13 ± 0.14^d^	7.55 ± 0.08^c^
RCP 2.6/2080	6.97 ± 0.19^b^	8.32 ± 0.16^b,c^	7.34 ± 0.15^c^	5.82 ± 0.18^c^	6.75 ± 0.14^c^	7.66 ± 0.15^b^	8.1 ± 0.08^cd^	7.60 ± 0.21^b^	6.79 ± 0.15^a^	7.61 ± 0.14^c^
RCP 4.5/2020	6.67 ± 0.11^a^	7.99 ± 0.15^a^	7.11 ± 0.07^b^	5.63 ± 0.09^b^	6.51 ± 0.07^b^	7.42 ± 0.07^a^	7.88 ± 0.12^b^	7.72 ± 0.10^b^	7.00 ± 0.10^c^	7.42 ± 0.09^b^
RCP 4.5/2050	7.15 ± 0.12^b,c^	8.42 ± 0.10^c^	7.80 ± 0.10^d^	5.95 ± 0.14^c,d^	6.85 ± 0.06^c,d^	7.83 ± 0.12^c^	8.27 ± 0.12^ed^	8.15 ± 0.22^d^	7.27 ± 0.14^e^	7.72 ± 0.10^d^
RCP 4.5/2080	7.42 ± 0.29^e^	8.7 ± 0.22^d^	7.70 ± 0.21^d^	6.23 ± 0.11 ^f^	7.11 ± 0.20^d^	8.11 ± 0.25^d^	8.51 ± 0.19^f^	8.49 ± 0.37^e^	7.19 ± 0.25	7.74 ± 0.26^d^
RCP 6.0/2020	6.65 ± 0.08^a^	7.99 ± 0.11^a^	7.11 ± 0.05^b^	5.59 ± 0.07^a^	6.49 ± 0.04^b^	7.44 ± 0.08^a,b^	7.90 ± 0.12^b^	7.71 ± 0.14^b^	6.96 ± 0.14^b^	7.38 ± 0.12^b^
RCP 6.0/2050	7.03 ± 0.11^b,c^	8.32 ± 0.10^b,c^	7.76 ± 0.11^d^	5.85 ± 0.10^c^	6.78 ± 0.09^c^	7.74 ± 0.11^b,c^	8.19 ± 0.11^d^	8.00 ± 0.07^c,d^	7.19 ± 0.10^d^	7.61 ± 0.06^c^
RCP 6.0/2080	7.18 ± 0.32^c,d^	8.3 ± 0.15^bc^	7.75 ± 0.16^d^	6.19 ± 0.21^f^	7.13 ± 0.15^d^	8.06 ± 0.20^d^	8.57 ± 0.17^f^	8.50 ± 0.21^f^	7.19 ± 0.21^d^	7.59 ± 0.15^c^
RCP 8.5/2020	6.65 ± 0.09^a^	8.00 ± 0.10^a^	7.10 ± 0.06^b^	5.62 ± 0.05^b^	8.19 ± 0.05^f^	7.44 ± 0.09^a,b^	7.88 ± 0.11^b^	7.77 ± 0.13^b,c^	6.97 ± 0.05^b,c^	7.40 ± 0.06^b^
RCP 8.5/2050	7.30 ± 0.14^d^	8.32 ± 0.10^b,c^	7.98 ± 0.16^e^	6.10 ± 0.14^e,f^	7.01 ± 0.14^d^	8.03 ± 0.17^d^	8.45 ± 0.15^f^	8.35 ± 0.25^e^	7.45 ± 0.15^f^	7.86 ± 0.11^d^
RCP 8.5/2080	8.01 ± 0.19^f^	8.91 ± 0.15^e^	8.33 ± 0.29^f^	6.83 ± 0.27^g^	7.69 ± 0.26^e^	8.89 ± 0.27^e^	9.22 ± 0.19^g^	9.29 ± 0.44^g^	8.21 ± 0.14^g^	8.07 ± 0.23^e^
SEm±	0.05	0.04	0.04	0.04	0.06	0.04	0.04	0.06	0.04	0.04
LSD (*P* = 0.05)	0.13	0.12	0.11	0.11	0.17	0.12	0.10	0.17	0.12	0.11
F calculated	49.48	56.82	88.39	56.28	25.43	63.92	102.25	83.66	53.59	56.05
Error degree of freedom	91	91	91	91	91	91	91	91	91	91

Value following different letter down the columns are significantly different using Tukey’s HSD test.

**Table 3:** Variation in number of generations (±standard deviation) of *Bactrocera zonata* on mango in four scenarios under future climate change periods.

Scenario/time period	Ranchi	Lucknow	Paria	Bengaluru	Vengurle	Sangareddy	Rewa	Rupnagar	Dharampuri	Mohanpur
Baseline	6.22^b^	6.75^a^	6.14^a^	5.39^c^	5.60^a^	6.64^a^	6.67^a^	5.64^a^	6.13^a^	6.30^a^
RCP 2.6/2020	5.99 ± 0.07^a^	7.18 ± 0.12^b^	6.40 ± 0.05^b^	4.99 ± 0.10^a^	5.95 ± 0.06^b^	6.72 ± 0.07^a,b^	7.14 ± 0.11^b^	6.98 ± 0.07^b^	6.26 ± 0.08^b^	6.66 ± 0.04^b^
RCP 2.6/2050	6.22 ± 0.10^b^	7.40 ± 0.13^c^	6.90 ± 0.11^d^	5.16 ± 0.13^b^	6.13 ± 0.10^c^	6.93 ± 0.13^b^	7.32 ± 0.14^c^	7.14 ± 0.13^c^	6.42 ± 0.13^c^	6.82 ± 0.08^c^
RCP 2.6/2080	6.27 ± 0.17^b^	7.54 ± 0.15^d^	6.62 ± 0.14^c^	5.18 ± 0.17^b^	6.06 ± 0.13^b,c^	6.83 ± 0.15^b^	7.33 ± 0.07^c^	6.88 ± 0.20^b^	6.12 ± 0.14^a^	6.87 ± 0.13^c^
RCP 4.5/2020	5.99 ± 0.11^a^	7.23 ± 0.14^b^	6.41 ± 0.07^b^	5.01 ± 0.08^a^	5.96 ± 0.06^b^	6.70 ± 0.07^a^	7.13 ± 0.11^b^	6.97 ± 0.09^b^	6.30 ± 0.10^b^	6.70 ± 0.08^b^
RCP 4.5/2050	6.44 ± 0.11^c^	7.58 ± 0.09^d^	7.05 ± 0.09^e^	5.31 ± 0.13^c^	6.26 ± 0.05^c^	7.08 ± 0.11^c^	7.50 ± 0.11^d^	7.39 ± 0.21^d^	6.55 ± 0.13^d^	6.98 ± 0.09^c,d^
RCP 4.5/2080	6.70 ± 0.28^d^	7.90 ± 0.21^e^	6.96 ± 0.19^d^	5.57 ± 0.10^d^	6.40 ± 0.19^d^	7.32 ± 0.24^d^	7.73 ± 0.18^e^	7.70 ± 0.34^e^	6.49 ± 0.24^c^	7.01 ± 0.40^d^
RCP 6.0/2020	5.97 ± 0.08^a^	7.15 ± 0.11^b^	6.40 ± 0.05^b^	4.97 ± 0.06^a^	5.95 ± 0.03^b^	6.71 ± 0.07^a,b^	7.15 ± 0.11^b^	6.97 ± 0.13^b^	6.26 ± 0.05^b^	6.65 ± 0.04^b^
RCP 6.0/2050	6.33 ± 0.10^b^	7.49 ± 0.09^c,d^	7.01 ± 0.08^d,e^	5.21 ± 0.09^b^	6.21 ± 0.10^c^	7.00 ± 0.10^c^	7.41 ± 0.11^c,d^	7.24 ± 0.07^c^	6.48 ± 0.10^c,d^	6.87 ± 0.06^c^
RCP 6.0/2080	6.47 ± 0.30^c,d^	7.54 ± 0.14^d^	7.00 ± 0.15^d,e^	5.54 ± 0.20^d^	6.42 ± 0.13^d^	7.24 ± 0.21^d^	7.78 ± 0.16^e,f^	7.71 ± 0.20^e^	6.49 ± 0.20^c,d^	6.87 ± 0.14^c^
RCP 8.5/2020	5.96 ± 0.09^a^	7.18 ± 0.10^b^	6.39 ± 0.06^b^	4.99 ± 0.05^a^	7.11 ± 0.05^e^	6.71 ± 0.08^a,b^	7.13 ± 0.11^b^	7.02 ± 0.12^b,c^	6.27 ± 0.05^b^	6.67 ± 0.06^b^
RCP 8.5/2050	6.58 ± 0.13^d^	7.49 ± 0.09^c,d^	7.22 ± 0.15^f^	5.45 ± 0.13^d^	6.42 ± 0.12^d^	7.27 ± 0.16^d^	7.66 ± 0.14^e^	7.57 ± 0.23^e^	6.72 ± 0.14^e^	7.11 ± 0.10^e^
RCP 8.5/2080	7.25 ± 0.18^e^	8.11 ± 0.14^f^	7.55 ± 0.27^g^	6.14 ± 0.25^e^	6.94 ± 0.24^e^	7.97 ± 0.30^e^	8.39 ± 0.18^g^	8.45 ± 0.41^f^	7.44 ± 0.13^f^	7.32 ± 0.21^f^
SEm±	0.04	0.04	0.04	0.03	0.05	0.04	0.04	0.06	0.04	0.04
LSD (*P* = 0.05)	0.12	0.11	0.10	0.10	0.13	0.12	0.10	0.16	0.11	0.12
F calculated	52.84	79.74	89.27	56.46	32.62	50.34	95.42	84.28	56.55	41.28
Error degree of freedom	91	91	91	91	91	91	91	91	91	91

Value following different letter down the column are significantly different using Tukey’s HSD test.

**Table 4:** Variation in number of generations (±standard deviation) of *Bactrocera correcta* on mango in four scenarios under future climate change periods.

Scenario/time period	Ranchi	Lucknow	Paria	Bengaluru	Vengurle	Sangareddy	Rewa	Rupnagar	Dharampuri	Mohanpur
Baseline	2.89^e^	2.77^a^	2.61^a^	2.65^e^	2.97^d^	2.35^b^	2.24^a^	2.33^a^	2.87 ^cd^	2.60^a^
RCP 2.6/2020	2.53 ± 0.04^a^	3.19 ± 0.06^a^	2.75 ± 0.02^b^	2.01 ± 0.05^a^	2.44 ± 0.03^a^	2.91 ± 0.03^c^	3.13 ± 0.06^b^	3.05 ± 0.03^b^	2.67 ± 0.04^a^	2.88 ± 0.02^b^
RCP 2.6/2050	2.65 ± 0.05^b^	3.30 ± 0.07^c^	3.01 ± 0.06^d^	2.10 ± 0.07^b^	2.55 ± 0.05^b^	3.02 ± 0.07^d^	3.23 ± 0.07^c^	3.14 ± 0.07^c^	2.76 ± 0.20^b^	2.96 ± 0.04^c^
RCP 2.6/2080	2.68 ± 0.09^b^	3.34 ± 0.07^c^	2.86 ± 0.07^c^	2.11 ± 0.09^b^	2.57 ± 0.06^b^	3.02 ± 0.07^d^	3.23 ± 0.03^c^	3.03 ± 0.10^b^	2.63 ± 0.07^a^	3.00 ± 0.06
RCP 4.5/2020	2.53 ± 0.05^a^	3.18 ± 0.07^a^	2.75 ± 0.03^b^	2.02 ± 0.04^a^	2.45 ± 0.03^a^	2.19 ± 0.04^a^	3.13 ± 0.06^b^	3.05 ± 0.05^b^	2.70 ± 0.07^ab^	2.90 ± 0.04^b,c^
RCP 4.5/2050	2.77 ± 0.06^d^	3.39 ± 0.05^d^	3.09 ± 0.05^e^	2.18 ± 0.06^c^	2.62 ± .03^b^	3.10 ± 0.06^e^	3.32 ± 0.06^d^	3.26 ± 0.11^d^	2.83 ± 0.07^c^	3.05 ± 0.05^d^
RCP 4.5/2080	2.90 ± 0.14^e,f^	3.53 ± .10^e^	3.04 ± .10^d^	2.31 ± 0.05^d^	2.75 ± 0.10^c^	3.24 ± 0.12^f^	3.44 ± 0.09^e^	3.43 ± 0.10^f^	2.82 ± 0.11^bc^	3.09 ± 0.21^d,e^
RCP 6.0/2020	2.52 ± 0.04^a^	3.18 ± 0.06^b^	2.75 ± 0.02^b^	2.0 ± 0.03^a^	2.44 ± 0.02^a^	2.91 ± 0.04^c^	3.14 ± 0.06^b^	3.05 ± 0.07^b^	2.67 ± 0.03^a^	2.88 ± 0.02^b^
RCP 6.0/2050	2.71 ± 0.05^c^	3.35 ± 0.05^c^	3.07 ± 0.04^e^	2.13 ± 0.05^c^	2.58 ± 0.05^b^	3.06 ± 0.05^d,e^	3.28 ± 0.06^c,d^	3.19 ± 0.04^c^	2.83 ± 0.05^c^	2.99 ± 0.03^c^
RCP 6.0/2080	2.78 ± 0.13^d^	3.37 ± 0.07^d^	3.06 ± 0.10^d,e^	2.30 ± 0.10^d^	2.75 ± 0.07^c^	3.21 ± 0.11^f^	3.47 ± 0.09^e,f^	3.43 ± 0.10^f^	2.82 ± 0.10^b,c^	3.02 ± 0.07^c,d^
RCP 8.5/2020	2.52 ± 0.05^a^	3.19 ± 0.05^b^	2.74 ± 0.03^b^	2.01 ± 0.03^a^	3.28 ± 0.03^e^	2.91 ± 0.04^c^	3.13 ± 0.05^b^	3.07 ± 0.06^b,c^	2.68 ± 0.03^a^	2.89 ± 0.03^b^
RCP 8.5/2050	2.84 ± 0.04^d,e^	3.35 ± 0.06^c,d^	3.18 ± 0.08^f^	2.25 ± 0.07^d^	2.70 ± 0.07^c^	3.20 ± 0.08^f^	3.41 ± 0.07^e^	3.36 ± 0.12^e^	2.91 ± 0.07^d^	3.12 ± 0.05^e^
RCP 8.5/2080	3.19 ± 0.11^g^	3.67 ± 0.07^f^	3.35 ± 0.14^g^	2.60 ± 0.13^e^	3.03 ± 0.12^d^	3.58 ± 0.13^g^	3.79 ± 0.09^g^	3.82 ± 0.21^g^	3.13 ± 0.06^e^	3.26 ± 0.11^f^
SEm±	0.02	0.02	0.02	0.02	0.03	0.02	0.02	0.03	0.03	0.02
LSD (*P* = 0.05)	0.06	0.06	0.05	0.05	0.08	0.06	0.05	0.08	0.07	0.06
F calculated	57.26	106.28	92.95	87.94	27.37	138.40	246.45	87.75	31.24	49.90
Error degree of freedom	91	91	91	91	91	91	91	91	91	91

Value following different letter down the column are significantly different using Tukey’s HSD test.

In the baseline period, number of generations of *B. correcta, B. zonata* and *B. dorsalis* were estimated to be 2.24–2.89; 5.6–6.75 and 6.03–8.28, respectively, which were predicted to be increased to 3.03–3.82; 6.14–8.45 and 6.83–9.29 numbers of generations, respectively under RCP 8.5 scenario during year 2080. In 2020, the gradual increase in voltinism was consistent among the four RCP scenarios. The number of all the fruit fly species generations increased during 2050 and more apparent increase was noticed in 2080 period. The present study also indicated that the maximum number of generations of each fruit fly species is expected to be in RCP 8.5 scenario towards the middle to northern part (Rupnagar, Lucknow, Rewa) of India. In RCP 8.5 and for the time period 2080, the lowest number of generations was predicted at Bengaluru for all the fruit fly species followed by Vengurle. Similar trends were found for all other scenarios.

**Figure 2:**
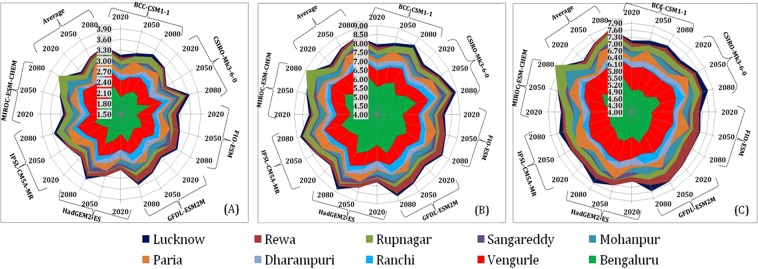
Inter model variation in number of generations of fruit flies (A = *B. correcta*; B = *B. dorsalis*; C = *B. zonata*) during future climate change periods across ten mango growing locations of India.

The location and time period wise variation in expected number of generations of each species as predicted for different GCM models is depicted (Fig. 2) where the numbers denotes the number of generations from centre to outer ring along with axis. Among three species *B. correcta* showed less number of generations and up to 8–9 generations are expected to occur in the future for *B. dorsalis* and *B. zonata*. The predictions for number of generations of each fruit fly species in all locations were higher for the CS model followed by Had model during each time period except Rupnagar location where maximum number of generations was expected during 2080 for MIR for all three species of fruit fly. Minimum number of generations was predicted from FI model for all the three fruit fly species for each study location in all the future time periods with only minor discrepancies.

### Variation in generation time and their relationship with voltinism

**Figure 3:**
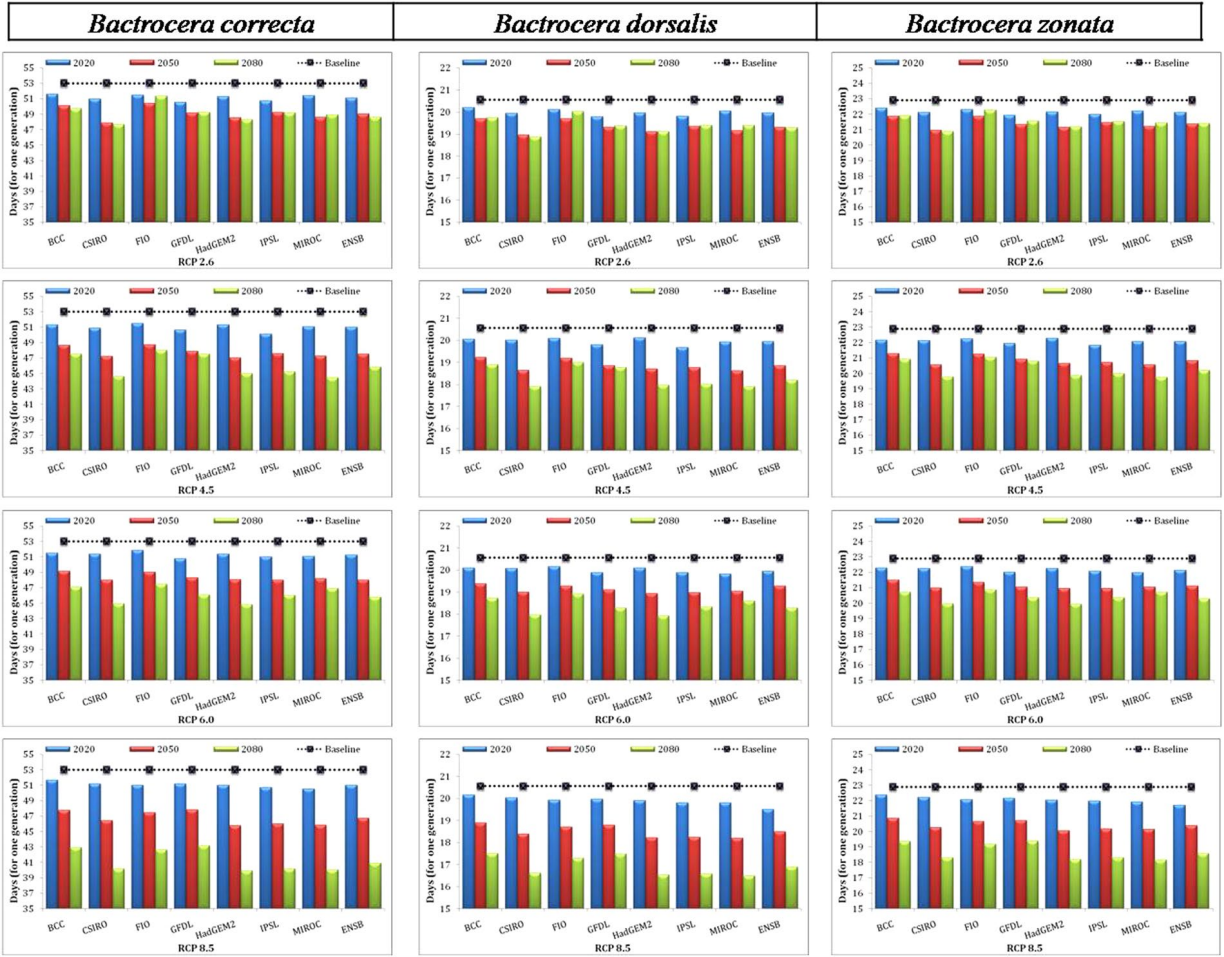
Inter-model and scenario variation in generation time of three major fruit flies species infesting mango fruits in India.

The range of generation time during the baseline period varies from 19.31 to 25.38, 20.74 to 25.97 and 47.66 to 61.69 days for *B. dorsalis, B. zonata* and *B. correcta*, respectively across different locations (Tables S2–S4). The results on changes in generation time during three future climate change periods using eight GCM models of the four scenarios (RCP 2.6, RCP 4.5, RCP 6.0 and RCP 8.5) are depicted in Fig. 3. The reduced generation time was predicted by majority of the models over the baseline period during future climate change periods. The highest change in mean generation time (0.90 to 23.52 days) was predicted for *B. correcta* in different model, time periods and scenarios (Fig. 3). The shortest generation time was predicted by IP model (very few for GF) and relative to longer generation time predicted by the FI in 2020 while in 2050 and 2080 time periods shortest time for generation was expected from CS and Had models in all scenarios for studied fruit fly species (Fig. 3). The longest generation time (in days) was predicted at Vengurle (60.38 to 36.17 ± 2.11) for *B. correcta* and *B. zonata* (24.59 to 16.68 ± 0.80) and *B. dorsalis* (22.44 to 15.21 ± 0.67) at Rupnagar during 2080 in RCP 8.5 scenario (Tables S2–S4).

The percent reduction in generation time over the baseline period across different model and scenarios after the mean of their respective all locations is presented in Table 5. The highest percent change (−2.74 to −24.70%) in generation time was predicted for *B. correcta* in different model, time periods and scenarios (Table 5). During the predicted future periods, highest percent change in generation time of fruit fly species was predicted for periods of 2080, followed by 2050 and 2020 over the baseline period across four scenarios and eight GCMs including ensemble (Table 5).

**Table 5:** Percent change in generation time of fruit flies during future climate change periods across different models and scenarios.

Scenario/time period	Fruit flies species	BCC-CSM1-1	CSIRO-Mk3-6-0	FIO-ESM	GFDL-ESM2M	HadGEM2-ES	IPSL-CM5A-MR	MIROC-ESM-CHEM	Ensemble
RCP 2.6/2020	*B. correcta*	−2.74	−3.87	−2.93	−4.74	−3.34	−4.35	−3.04	−3.70
	*B. zonata*	−2.26	−3.42	−2.67	−4.24	−3.31	−3.92	−3.00	−3.42
	*B. dorsalis*	−1.90	−3.21	−2.40	−3.95	−3.14	−3.87	−2.67	−3.16
RCP 2.6/2050	*B. correcta*	−5.57	−9.75	−4.98	−7.29	−8.49	−7.16	−8.31	−7.55
	*B. zonata*	−4.56	−8.52	−4.56	−6.86	−7.64	−6.26	−7.44	−6.66
	*B. dorsalis*	−4.34	−8.00	−4.34	−6.31	−7.20	−6.07	−7.03	−6.27
RCP 2.6/2080	*B. correcta*	−6.19	−10.05	−3.13	−7.22	−8.88	−7.28	−7.72	−8.30
	*B. zonata*	−4.34	−8.82	−2.78	−5.89	−7.50	−6.07	−6.49	−6.51
	*B. dorsalis*	−4.14	−8.44	−2.85	−5.95	−7.20	−5.90	−6.55	−6.42
RCP 4.5/2020	*B. correcta*	−3.30	−4.01	−2.93	−4.53	−3.27	−5.53	−3.77	−3.85
	*B. zonata*	−3.28	−3.37	−2.79	−4.22	−2.73	−4.68	−3.64	−3.61
	*B. dorsalis*	−2.62	−2.89	−2.47	−3.89	−2.33	−4.49	−3.26	−3.15
RCP 4.5/2050	*B. correcta*	−8.26	−10.99	−8.16	−9.79	−11.28	−10.33	−10.89	−10.39
	*B. zonata*	−7.10	−10.21	−7.21	−8.62	−9.84	−9.61	−10.28	−9.08
	*B. dorsalis*	−6.66	−9.50	−6.88	−8.50	−9.23	−8.94	−9.60	−8.54
RCP 4.5/2080	*B. correcta*	−10.36	−15.86	−9.47	−10.46	−15.22	−14.74	−16.21	−13.60
	*B. zonata*	−8.62	−13.57	−8.05	−9.22	−13.21	−12.77	−13.76	−11.80
	*B. dorsalis*	−8.27	−13.14	−7.81	−8.87	−12.80	−12.58	−13.10	−11.69
RCP 6.0/2020	*B. correcta*	−2.92	−3.17	−2.29	−4.23	−3.15	−3.82	−3.69	−3.43
	*B. zonata*	−2.79	−2.83	−2.29	−3.86	−2.91	−3.61	−4.09	−3.31
	*B. dorsalis*	−2.41	−2.54	−2.12	−3.50	−2.50	−3.53	−3.81	−3.22
RCP 6.0/2050	*B. correcta*	−7.30	−9.51	−7.63	−8.92	−9.34	−9.47	−9.21	−9.52
	*B. zonata*	−6.16	−8.31	−6.78	−8.07	−8.53	−8.44	−8.17	−7.85
	*B. dorsalis*	−5.97	−7.82	−6.45	−7.32	−8.06	−7.91	−7.61	−6.44
RCP 6.0/2080	*B. correcta*	−11.18	−15.26	−10.49	−13.09	−15.51	−13.24	−11.50	−13.71
	*B. zonata*	−9.60	−12.82	−8.90	−11.14	−13.00	−11.18	−9.57	−11.42
	*B. dorsalis*	−9.14	−12.79	−8.21	−11.28	−12.98	−11.05	−9.69	−11.26
RCP 8.5/2020	*B. correcta*	−2.61	−3.49	−3.84	−3.45	−3.85	−4.42	−4.78	−3.83
	*B. zonata*	−2.34	−2.97	−3.60	−3.25	−3.71	−4.07	−4.27	−5.21
	*B. dorsalis*	−2.15	−2.74	−3.25	−3.07	−3.42	−3.84	−3.90	−5.33
RCP 8.5/2050	*B. correcta*	−10.01	−12.43	−10.55	−9.89	−13.67	−13.31	−13.57	−11.84
	*B. zonata*	−8.87	−11.49	−9.81	−9.53	−12.39	−11.99	−12.07	−11.02
	*B. dorsalis*	−8.33	−10.75	−9.25	−8.84	−11.58	−11.50	−11.66	−10.28
RCP 8.5/2080	*B. correcta*	−19.08	−24.23	−19.67	−18.56	−24.70	−24.32	−24.47	−22.92
	*B. zonata*	−15.42	−20.10	−16.23	−15.31	−20.61	−20.14	−20.76	−18.84
	*B. dorsalis*	−15.09	−19.35	−16.09	−15.16	−19.79	−19.51	−19.95	−18.01

To examine the relationship between voltinism and generation time, the data of generation time was plotted against number of generations for the three climate change periods (2020, 2050 and 2080), four (RCP 2.6, RCP 4.5, RCP 6.0 and RCP 8.5) green house gas concentration scenarios and across eight models (Fig. S1). We also performed the liner regression analysis by using data of generation time and voltinism for generating statistical inferences. The number of fruit flies generations was estimated to be significantly (P < 0.001) increased by 2080 under the four RCP scenarios with reduced generation time (Fig. S1). A significant linear relation was observed for *B. correcta* (*P* < 0.001; *F* = 36.67) and *B. dorsalis* (*P* < 0.001; *F* = 1477.30) during 2050 but was non-significant for *B. zonata* (*P* = 0.02; *F* = 5.83). The relationships among three species of fruit fly indicate the species specificity and same is reflected with *B. zonata*. Further, some of the deviations and discrepancies in the temperature data set across four RCP scenarios might have reflected in the differential response among three species of fruit fly. Significant linear relations among values indicate the possibility of occurrence of higher number of generations with reduced generation time. The advancement of growth stages of insect pest due to increased temperature lead to hastening of life cycle and reduction of generation time which is reflected in occurrence of more number of generations of three fruit fly species. The rate of increase of generations is higher (ranged from 0–2 generations) with RCP 8.5 during different time periods and manifested more in 2080 s climate change period (1–2 generations) with proportional reduction of generation time.

### Partitioned variation

**Figure 4:**
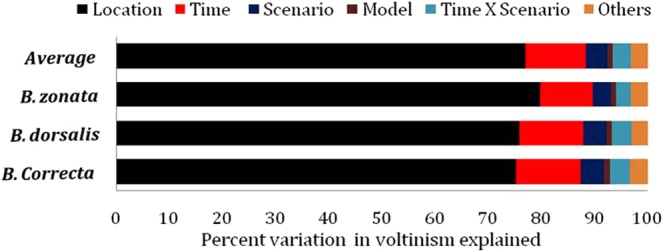
The relative degree of various sources of uncertainty in the predicted voltinism of three *Bactrocera* species. In bars others represents all remaining interactions except time × scenario interaction.

Variance partitioning for the predicted increases in generations were determined and observed the similar trend for all the investigated fruit fly species (Fig. 4). The average of the sources are as follows; locations (76.9%), time periods (11.3%), scenario (4.1%) and the time period × scenario interaction (3.5%) which altogether explained up to 95.8% of the total variation. The others (models and interaction among variables) accounted for minor part of the total variation. The observed percentages of variation were more or less similar across the investigated three species of fruit fly.

### Estimation of YIR (Yield Infestation Relationships) during future climate change periods

The linear equation proposed for estimating the mango fruit infestation and the number of generations was found to be significant (*P* < 0.01; *F* = 32.35) (Table 6). The trend and range of variations in estimated and projected infestation of mango fruits are similar among majority of locations tested except for Rupnagar. Under current climatic conditions, fruit flies can cause an average estimated infestation of 28.8% in mango cultivated in India. These yield infestations are expected to increase to31.3, 34.2 and 36.1% of the total mango production leading to an average increase in infestation by 2.4, 5.3 and 7.2% in 2020, 2050 and 2080, respectively due to increased number of generations (Table 6). The predicted increase in average mango fruit infestation due to fruit flies is much higher in the Northern locations of India (Rupnagar, Lucknow) as compared to other locations.

**Table 6:** Predicted mango fruit infestation (%) at selected locations of India due to fruit flies infestation during current and future climatic conditions.

Location/s	Estimated fruit infestation due to fruit flies (%)*	Generation load of fruit flies^#^				Predicted fruit infestation				Fruit infestation difference over current infestation		
		1969–2005	2020	2050	2080	1969–2005	2020	2050	2080	2020	2050	2080
Lucknow	38.00 ± 1.32	17.80	18.37	19.16	19.81	36.07	38.00	40.70	42.89	1.93	4.63	6.82
Paria	25.16 ± 2.63	15.58	16.25	17.92	17.89	28.52	30.81	36.50	36.37	2.29	7.98	7.85
Rewa	32.12 ± 1.26	16.31	18.15	19.03	19.89	31.00	37.29	40.25	43.17	6.29	9.25	12.17
Vengurle	18.50 ± 0.00	14.81	15.81	15.70	16.40	25.90	29.32	28.94	31.31	3.42	3.04	5.41
Rupnagar	40.25 ± 7.24	14.27	17.77	18.67	19.58	24.06	35.97	39.03	42.13	11.91	14.97	18.07
Sangareddy	29.79 ± 2.08	16.35	17.05	17.98	18.78	31.14	33.53	36.69	39.41	2.39	5.55	8.27
Ranchi	36.17 ± 5.53	16.03	15.16	16.23	16.96	30.05	27.10	30.75	33.19	−2.95	0.7	3.14
Bengaluru	16.50 ± 5.73	14.07	12.61	13.37	14.20	23.38	18.43	21.08	23.84	−4.95	−2.3	0.46
Mohanpur	28.35 ± 2.07	15.90	15.92	17.66	17.86	29.61	33.19	35.59	36.28	3.58	5.98	6.67
Dharampuri	24.52 ± 0.00	15.82	16.95	16.64	16.83	29.33	29.69	32.11	32.72	0.36	2.78	3.39
**Average**	**28.85**	**15.69**	**16.40**	**17.23**	**17.82**	**28.90**	**31.33**	**34.16**	**36.13**	**2.43**	**5.26**	**7.23**

*Fruit infestation estimated from different published literature which depends on varieties and fruit ripening season.

^#^Generation of fruit flies estimated only for mango fruiting season.

## Discussion

In present study, all the seven GCM models and the ensemble used predicted an increasing trend in temperature projections during future climate change periods in all the scenarios. It is expected that maximum temperature would fluctuate by ±0.47 to ±4.02 °C and minimum temperature by ±0.43 to ±6.78 °C during future climate change periods over the baseline of four scenarios (RCP 2.6, RCP 4.5, RCP 6.0 and RCP 8.5) at ten locations of India. It is understood that the mean temperature at majority of locations would increase significantly during three future climate change periods. Increasing trends of temperature were also reported in different locations of India using MarkSim GCM multimodal data by Srinivasa Rao *et al*.^6,8^ for the period of 2080. Chaturvedi *et al*.^44^ mentioned that mean warming in India is likely to be in the range of 1.7–2 °C by 2030 s and 3.3–4.8 °C by 2080 s relative to preindustrial times. In present study, the entire seven models also indicated similar warmer climate for the future time periods. These changes in temperature have the potential to influence the distribution and abundance of insect pests in different parts of world^45^. These influences urged the scientific communities to improve understanding of location specific dynamics of a particular insect pest species in relation to a specific crop under changed climate scenarios.

Shorter development times leading to increased number of generations have been predicted in multivoltine arthropods under changed climatic conditions^46^. Consistent with these global trends, the present study identified significant changes in number of generations of fruit flies as a consequence of changing climate in Indian mango growing locations. Voltinism of three fruit fly species estimated with the daily mean surface air temperature from the MarkSim multimodal data simulations were consistent with the reports that higher temperature resulted in additional generations of multivoltine insect species^6,8,13,47,48^. Temperature projections indicated that 1–2 additional generations would occur during 2050 and 2080 due to higher temperature projected in CS and Had models. The temperature projections of these models found that the baseline generation time i.e. 19.31 to 25.38, 20.74 to 25.97 and 47.66 to 61.69 days of *B. dorsalis, B. zonata* and *B. correcta*, respectively on mango would decrease by 15–24% during future climate change periods. These results were consistent with earlier GDD based multimodal data predictions indicating that more number of generations were expected to occur during future climate change periods^49^ indicating that the insects respond to higher temperature with increased rates of development and more generations with less generation time. However, extra generations is only a roust conclusion at the end of centaury but it will also depend on how agricultural practices change in response to the changing climate^13^.

Location based difference in number of generation and generation time in India predominately reported in case of *Spodoptera litura* on peanut^8^ and *H. armigera* on pigeon pea crop^6^. On the similar lines, in the present study, the predicted number of generations also varies significantly among the locations and was found to be higher at northern locations i.e. Rupnagar and Lucknow whereas minimum numbers at Bengaluru across four scenarios. The more number of generations in northern India locations was the consequences of maximum predicted increase in minimum and maximum temperature during future climate change scenarios. The previous results showed the uncertainties in degree days based approaches related from locations^6,8^, time periods^7^, model selection^13^ and above all in scenarios where we used RCPs in present study. Considering these points, we used ANOVA to partition the variation in the resulting voltinism among time period, climate change scenario, GCM and geographical location. For the three species of the fruit fly, the geographical location explained an average of 77% of the total variation in voltinism, far more than the time period (11%) or scenarios (4%). The interaction between time periods and scenarios explained the 3% variation. These results are significant in the sense that global warming is a spatio-temporally connected process^15^ i.e. spatial (locations) followed by temporal (time periods). Higher variation in voltinism due to geographical location and time period, as observed in present study, is well supported by earlier studies by Srinivasa Rao *et al*.^8^ and Srinivasa Rao *et al*.^6^ for Indian region. The locations selected in the present study are situated in different agro-climatic regions of the country. These climates range from hot semi-arid (Dharmpuri) to hot sub-humid (Lucknow) ecoregions. Since, temperature is major factor that governs the insect phenology, it is inherent that geographical locations having diverse climatic conditions and ecologies will lead to more variation in the voltinism. Unexpectedly, variation due to model selection was found very low in contrast to earlier study^13^. This may be due to reduction in the uncertainties by the selection of more number of GCM models (8) in present study as compared to three GCMs selected by Ziter *et al*.^13^. Poor understanding and difficulties involved in predicting the future changes, inclusion of multiple GCM-scenario combinations is always must to overcome the uncertainties from the GCMs and scenarios for biological impact studies^7,13,50^.

Based on the relationship established between fruit flies numbers and estimated fruit infestation from various literature reports, these three species of fruit flies can cause an average of 28.8% (range 16.5–40.2%) fruit infestation. These predictions are in agreement with earlier reports on mango infestation due to fruit flies i.e. 21.6–58.3% fruit infestation across various locations of India. Under current climatic conditions, fruit flies can cause an average estimated infestation of 28.8% and are expected to increase to 31.3, 34.2 and 36.1% of the total mango production leading to an average increase in infestation by 2.4, 5.3 and 7.2% in 2020, 2050 and 2080, respectively. The predicted increase in average mango fruit infestation due to fruit flies is much higher in the Northern locations of India (Rupnagar, Lucknow) as compared to other locations during future climate change periods due to additional 1–2 generations. There was a little deviation between observed and estimated fruit fly infestation at Rupnagar. It may be due to limited (one time observation) fruit infestation data from these locations. It would always be better if more number of observations from different years is available for representation of yield losses in particular area^43^. In spite of this sound linear relationship was established between generation numbers and estimated fruit infestation. To overcome the limitations in degree days based approach predictions, we selected maximum number of locations representing whole mango growing region, a sufficient numbers of GCM models with all four scenarios (RCPs) as suggested by earlier workers^7,13^ but still it has some limitations i.e. present study totally depend on temperature whereas other factors such as precipitation, moisture, elevated CO_2_, crop phenology, and multi-species interactions including pest-parasitoid also should be considered under climate change scenario related studies. In addition to temperature, soil moisture level can be an important factor that affects soil pupating fruit flies under future climate change^51^. To achieve more comprehensive results, studies of climate change effects on changes in soil moisture will also help to understand population dynamics of the fruit flies in the future. In spite of the current study’s limitations, present study findings indicate the possibility of occurrence of more number of generations of mango fruit fly species with reduced generation time. These results imply that probability of more incidence of fruit fly in majority of mango growing locations of the country. As it is well known that incidence is the function of number of generations of insect pest though further investigations are required to quantify exactly the relationships. Insect species with reduced generation time and higher population can produce significant changes on their population dynamics. Insect mortality may also decrease with warmer temperatures and in turn more population is expected.

## Conclusion

The number of generations of multivoltine insect species, Fruit fly *Bactrocera* sp. is likely to increase during future climate change periods across four RCPs and at majority of mango growing locations of India. Such increases can greatly exacerbate the pest incidence. Increase in mean surface temperature of >2–4 °C can have dramatic effects on insect voltinism by causing shift in the development period. Our studies indicated that possibility of occurrence of more number of generations of *Bactrocera* sp. on mango at majority of locations during future climate change periods across four climate change emission scenarios. The present studies were conducted considering the increase in temperature alone due to the paucity of available information albeit other factors also influence the same. In addition to the present data availability on increase in temperature and further data access to variation of CO_2_ levels across scenarios, models and locations will give the complete understanding of pest dynamics during future climate change periods. Further, the effects of climate change on insect pests are of confounding and complex in nature and the comprehension of these complex associations is possible only when complete information is available. Regardless of the current study’s limitations, it would be prudent to assess whether current fruit fly management practices in high risk mango production areas (i.e. Rupnagar and Lucknow) would remain effective under projected temperature increases.

## Supplementary information

Supplementary information's

## Data Availability

All the weather data of each location are freely accessible via http://gisweb.ciat.cgiar.org/MarkSimGCM/. Degree days approaches processed data available on request from authors.
