# Supplementary material for: Spatio-temporal temperature variations in MarkSim multimodel data and their impact on voltinism of fruit fly, Bactrocera species on mango
Source: Sci Rep. 2019 Jul 4;9:9708. doi: 10.1038/s41598-019-45801-z (PMC6609607; doi:10.1038/s41598-019-45801-z)
Supplement: Supplementary file 1 — Supplementary information's [file 41598_2019_45801_MOESM1_ESM.doc]

**Spatio-temporal temperature variations in MarkSim multimodel data and their impact on voltinism of fruit fly, *Bactrocera* species on mango**

Jaipal Singh Choudhary^1*^, Santosh S. Mali^1^, Debu Mukherjee^1^, Anjali Kumari^1^, Moanaro L^1^,

M. Srinivasa Rao^2^, Bikash Das^1^, A.K. Singh^1^ and B.P. Bhatt^3^

**Supplementary Information**


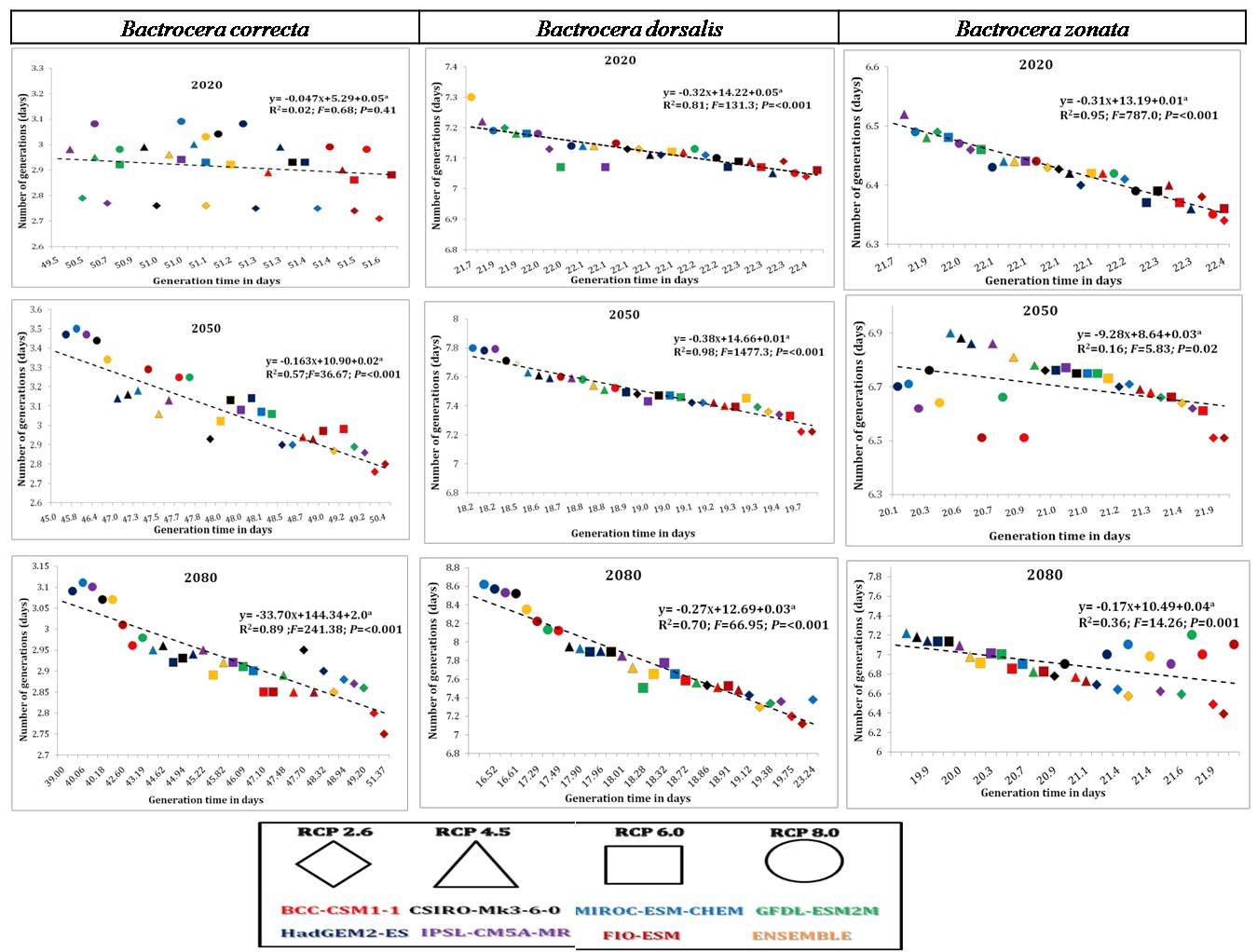


**Figure S1. Relationship between predicted number of generations and generation time of *Bactrocera* sp across three climate change periods, four scenarios and eight models**. ^a^values denotes constant estimated from the data; ^a^ random error term

**Supplementary Table S1:** **Comparison of the mean of minimum and maximum daily temperature projections between seven GCMs models and their average across different locations of mango growing regions.**

| Locations | Year | 2020 | | | | | | | | 2050 | | | | | | | | 2080 | | | | | | | |
| --- | --- | --- | --- | --- | --- | --- | --- | --- | --- | --- | --- | --- | --- | --- | --- | --- | --- | --- | --- | --- | --- | --- | --- | --- | --- |
|  | Model | GF | FI | CS | BC | Had | IP | MI | ENS | GF | FI | CS | BC | Had | IP | MI | ENS | GF | FI | CS | BC | Had | IP | MI | ENS |
|  | Tem. |  |  |  |  |  |  |  |  |  |  |  |  |  |  |  |  |  |  |  |  |  |  |  |  |
| Lucknow | Max | 32.98 | 32.9 | 32.68 | 32.82 | 32.22 | 33.02 | 32.72 | 32.77 | 33.62 | 33.42 | 33.56 | 33.25 | 33.27 | 33.65 | 33.35 | 33.46 | 34.58 | 34.31 | 34.77 | 34.35 | 34.77 | 35.1 | 34.20 | 34.65 |
|  | Min | 20.32 | 20.15 | 20.31 | 20.03 | 20.16 | 20.3 | 20.18 | 20.21 | 20.86 | 20.59 | 21.45 | 20.46 | 21 | 21.31 | 20.68 | 20.95 | 21.54 | 21.46 | 22.99 | 21.54 | 22.29 | 22.47 | 21.98 | 22.05 |
| Mohanpur | Max | 31.78 | 31.68 | 31.71 | 31.67 | 31.55 | 31.84 | 31.67 | 31.71 | 32.37 | 32.23 | 32.63 | 32.18 | 32.47 | 32.68 | 32.31 | 32.43 | 33.05 | 32.64 | 33.57 | 32.77 | 33.57 | 33.6 | 32.95 | 33.20 |
|  | Min | 22.96 | 22.81 | 22.89 | 22.85 | 22.94 | 23.09 | 22.85 | 22.92 | 23.48 | 23.36 | 23.9 | 23.48 | 23.94 | 23.99 | 23.56 | 23.69 | 24.07 | 23.79 | 24.92 | 24.11 | 24.94 | 24.83 | 24.50 | 24.44 |
| Paria | Max | 32.61 | 32.64 | 32.5 | 32.67 | 32.71 | 32.57 | 32.60 | 32.62 | 34.61 | 34.73 | 34.95 | 34.67 | 35.2 | 34.72 | 34.69 | 34.81 | 33.69 | 33.77 | 34.44 | 33.73 | 34.64 | 34.15 | 33.97 | 34.07 |
|  | Min | 22.53 | 22.47 | 22.72 | 22.58 | 22.67 | 23.02 | 22.61 | 22.67 | 22.83 | 22.72 | 23.68 | 22.88 | 23.46 | 23.8 | 23.10 | 23.23 | 23.88 | 23.67 | 25.34 | 23.93 | 24.82 | 25.2 | 24.35 | 24.47 |
| Ranchi | Max | 29.92 | 29.78 | 29.68 | 29.88 | 29.46 | 30.05 | 29.76 | 29.80 | 30.77 | 30.64 | 30.86 | 30.38 | 30.76 | 30.86 | 30.25 | 30.71 | 31.53 | 31.08 | 32.01 | 31.17 | 31.99 | 31.56 | 31.45 | 31.56 |
|  | Min | 18.72 | 18.52 | 18.73 | 18.61 | 18.6 | 19.0 | 18.62 | 18.70 | 19.55 | 19.39 | 20.15 | 19.29 | 20.03 | 20.51 | 19.70 | 19.82 | 20.24 | 19.87 | 21.5 | 20.17 | 21.14 | 21.1 | 2.54 | 20.67 |
| Rewa | Max | 32.62 | 32.51 | 32.22 | 32.38 | 31.78 | 32.56 | 32.20 | 32.35 | 33.3 | 33.19 | 33.21 | 32.87 | 32.99 | 33.29 | 32.98 | 33.14 | 34.07 | 33.74 | 34.17 | 33.82 | 33.98 | 34.44 | 33.85 | 34.04 |
|  | Min | 19.92 | 19.64 | 19.83 | 19.49 | 19.63 | 20.24 | 19.63 | 19.79 | 20.62 | 20.29 | 21.1 | 20.03 | 20.81 | 21.32 | 20.35 | 20.70 | 21.48 | 20.88 | 22.36 | 21.07 | 21.73 | 22.29 | 21.63 | 21.64 |
| Rupnagar | Max | 32.03 | 31.84 | 31.97 | 31.92 | 31.62 | 32.09 | 31.72 | 31.91 | 32.6 | 32.48 | 33.11 | 32.89 | 32.86 | 33.12 | 32.85 | 32.84 | 33.24 | 33.07 | 34.23 | 33.79 | 34.07 | 34.33 | 33.85 | 33.79 |
|  | Min | 18.54 | 18.46 | 18.76 | 18.5 | 18.48 | 18.99 | 18.52 | 18.62 | 19.33 | 19.09 | 20 | 19.35 | 19.48 | 20.24 | 19.45 | 19.58 | 19.95 | 19.54 | 21.28 | 20.21 | 20.52 | 21.55 | 20.58 | 20.51 |
| Bengaluru | Max | 29.8 | 29.66 | 29.68 | 29.66 | 29.82 | 29.8 | 29.56 | 29.74 | 30.23 | 30.22 | 30.55 | 30.17 | 30.74 | 30.45 | 30.40 | 30.39 | 31.06 | 30.89 | 31.45 | 30.87 | 31.65 | 31.27 | 30.65 | 31.20 |
|  | Min | 18.94 | 18.63 | 18.71 | 18.82 | 18.97 | 19.0 | 18.62 | 18.85 | 19.42 | 19.18 | 19.88 | 19.68 | 20.12 | 19.95 | 19.56 | 19.71 | 20.23 | 19.81 | 20.91 | 20.37 | 21.1 | 20.88 | 20.61 | 20.55 |
| Vengurle | Max | 31.67 | 31.75 | 29.65 | 31.78 | 31.89 | 31.86 | 31.35 | 31.43 | 32.08 | 32.36 | 32.6 | 32.35 | 32.77 | 32.65 | 32.51 | 32.47 | 32.7 | 32.82 | 33.47 | 32.86 | 33.52 | 33.46 | 33.20 | 33.14 |
|  | Min | 23.51 | 23.35 | 23.45 | 23.42 | 23.54 | 23.82 | 23.41 | 23.52 | 24.15 | 23.96 | 26.44 | 24.1 | 24.46 | 24.82 | 24.50 | 24.66 | 24.67 | 24.39 | 25.38 | 24.69 | 25.5 | 25.85 | 24.95 | 25.08 |
| Sangareddy | Max | 32.85 | 32.84 | 32.66 | 32.64 | 32.77 | 32.79 | 32.63 | 32.76 | 33.35 | 33.49 | 33.5 | 33.19 | 33.83 | 33.57 | 33.35 | 33.49 | 34.14 | 34.05 | 34.07 | 34.41 | 33.89 | 34.74 | 34.56 | 34.22 |
|  | Min | 21.8 | 21.69 | 21.77 | 21.61 | 21.87 | 22.07 | 21.65 | 21.80 | 22.4 | 22.33 | 22.89 | 22.38 | 23.13 | 23.11 | 22.68 | 22.71 | 22.86 | 23.06 | 23.06 | 24.09 | 23.21 | 24.21 | 23.20 | 23.42 |
| Dharampuri | Max | 32.84 | 32.54 | 32.62 | 32.55 | 32.66 | 32.68 | 32.42 | 32.65 | 33.26 | 33.05 | 33.47 | 33.07 | 33.63 | 33.33 | 33.15 | 33.30 | 33.99 | 33.77 | 34.41 | 33.8 | 34.47 | 34.14 | 33.98 | 34.10 |
|  | Min | 22.2 | 21.87 | 22.06 | 22.09 | 22.25 | 22.23 | 22.10 | 22.12 | 22.66 | 22.41 | 23.09 | 22.88 | 23.37 | 23.13 | 22.65 | 22.92 | 23.28 | 23.04 | 24.1 | 23.54 | 24.13 | 24.11 | 23.65 | 23.70 |

Here model GFDL-ESM2M (GF); FIO-ESM (FI); CSIRO-Mk3-6-0(CS); BCC-CSM1-1(BC); HadGEM2-ES (Had); IPSL-CM5A-MR (IP); MIROC-ESM-CHEM (MI) and ensemble of all (ENS) refers in table

**Supplementary Table S2. Variation in generation time of *Bactrocera dorsalis*** on mango in four scenarios under future climate change periods.

| Scenario/time period | Ranchi | Lucknow | Paria | Bengaluru | Vengurle | Sangareddy | Rewa | Rupnagar | Dharampuri | Mohanpur |
| --- | --- | --- | --- | --- | --- | --- | --- | --- | --- | --- |
| Baseline | 21.46 | 19.31 | 20.64 | 25.38 | 23.63 | 20.96 | 19.98 | 22.44 | 22.59 | 21.2 |
| RCP 2.6/2020 | 23.46±0.27 | 19.6± 0.31 | 22.01±0.22 | 28.05±0.56 | 23.66±0.24 | 20.87±0.16 | 19.66±0.32 | 20.23±0.20 | 22.51±0.31 | 21.11±0.16 |
| RCP 2.6/2050 | 20.32±0.31 | 17.15±0.30 | 18.31±0.27 | 24.33±0.61 | 20.97±0.46 | 18.31±0.26 | 17.43±0.32 | 17.84±0.31 | 19.76±0.41 | 18.67±0.22 |
| RCP 2.6/2080 | 20.05±0.50 | 16.96±0.34 | 19.18±0.42 | 24.22±0.85 | 20.69± | 18.35±0.32 | 17.4±0.17 | 17.68±0.50 | 19.75±0.42 | 18.5±0.35 |
| RCP 4.5/2020 | 23.48±0.40 | 19.47±0.39 | 21.93±0.25 | 27.9±0.44 | 23.55±0.21 | 21.11±0.16 | 19.75±0.31 | 20.22±0.25 | 22.34±0.36 | 20.98±0.23 |
| RCP 4.5/2050 | 19.69±0.33 | 16.71±0.22 | 18.06±0.23 | 23.56±0.54 | 20.52±0.13 | 18±0.28 | 16.98±0.39 | 17.33±0.47 | 19.37±0.40 | 18.19±0.24 |
| RCP 4.5/2080 | 18.89±0.81 | 16.08±0.40 | 18.2±0.47 | 22.54±0.50 | 19.85±0.52 | 17.35±0.58 | 16.43±0.39 | 16.61±0.72 | 18.59±0.58 | 17.37±0.85 |
| RCP 6.0/2020 | 23.51±0.29 | 19.66±0.30 | 21.99±0.21 | 28.2±0.36 | 23.65±0.11 | 20.91±0.24 | 19.64±0.31 | 20.24±0.38 | 22.49±0.21 | 21.12±0.14 |
| RCP 6.0/2050 | 20±0.30 | 16.93±0.21 | 18.11±0.23 | 24.05±0.48 | 22.65±0.34 | 18.11±0.19 | 17.17±0.26 | 17.63±0.15 | 19.58±0.29 | 18.51±0.17 |
| RCP 6.0/2080 | 19.57±0.95 | 16.05±0.27 | 18.1±0.35 | 22.65±0.79 | 19.83±0.36 | 17.49±0.50 | 16.24±0.37 | 16.61±0.45 | 18.57±0.52 | 17.69±0.32 |
| RCP 8.5/2020 | 23.48±0.34 | 19.59±0.25 | 22.02±0.22 | 27.99±0.30 | 19.79±0.19 | 20.91±0.24 | 19.71±0.33 | 20.07±0.35 | 22.48±0.17 | 21.03±0.20 |
| RCP 8.5/2050 | 19.24±0.40 | 16.93±0.21 | 17.65±0.39 | 23.06±0.50 | 20.13±0.37 | 17.54±0.44 | 16.58±0.34 | 16.91±0.52 | 18.85±0.35 | 17.93±0.23 |
| RCP 8.5/2080 | 17.59±0.55 | 15.04±0.31 | 16.91±0.64 | 20.66±0.74 | 18.39±0.66 | 16.01±0.44 | 15.26±0.37 | 15.21±0.67 | 17.19±0.29 | 16.64±0.48 |

**Supplementary Table S3. Variation in generation time of *Bactrocera zonata*** on mango in four scenarios under future climate change periods.

| Scenario/time period | Ranchi | Lucknow | Paria | Bengaluru | Vengurle | Sangareddy | Rewa | Rupnagar | Dharampuri | Mohanpur |
| --- | --- | --- | --- | --- | --- | --- | --- | --- | --- | --- |
| Baseline | 22.63 | 20.75 | 22.96 | 25.97 | 24.82 | 20.74 | 21.12 | 24.59 | 23 | 22.39 |
| RCP 2.6/2020 | 23.46±0.27 | 19.6±0.31 | 22.01±0.22 | 28.05±0.56 | 23.66±0.24 | 20.87±0.16 | 19.66±0.32 | 20.23±0.20 | 22.51±0.31 | 21.11±0.16 |
| RCP 2.6/2050 | 22.54±0.45 | 18.97±0.36 | 20.4±0.31 | 27.12±0.71 | 22.96±0.36 | 20.28±0.38 | 19.18±0.42 | 19.73±0.36 | 21.86±0.51 | 20.71±0.23 |
| RCP 2.6/2080 | 22.36±0.70 | 18.59±0.43 | 21.2±0.50 | 27.05±0.93 | 23.21±0.45 | 20.66±0.59 | 19.1±0.21 | 19.55±0.50 | 21.83±0.56 | 20.54±0.37 |
| RCP 4.5/2020 | 23.48±0.40 | 19.47±0.39 | 21.93±0.25 | 27.9±0.44 | 23.55±0.21 | 21.11±0.16 | 19.75±0.31 | 20.22±0.25 | 22.34±0.36 | 20.98±0.23 |
| RCP 4.5/2050 | 21.68±0.45 | 18.44±0.28 | 20±0.25 | 26.4±0.65 | 22.56±0.18 | 19.89±0.36 | 18.61±0.30 | 19.11±0.59 | 21.34±0.42 | 20.17±0.27 |
| RCP 4.5/2080 | 21.01±0.84 | 17.85±0.43 | 20.28±0.55 | 25.05±0.55 | 22.12±0.65 | 19.31±0.58 | 18.19±0.37 | 18.27±0.88 | 20.73±0.61 | 19.16±0.98 |
| RCP 6.0/2020 | 23.51±0.29 | 19.66±0.30 | 21.99±0.21 | 28.2±0.36 | 23.65±0.11 | 20.91±0.24 | 19.64±0.31 | 20.24±0.38 | 22.49±0.21 | 21.12±0.14 |
| RCP 6.0/2050 | 22.12±0.39 | 18.72±0.27 | 20.12±0.22 | 26.83±0.47 | 22.71±0.37 | 20.07±0.29 | 18.84±0.31 | 19.5±0.18 | 21.61±0.36 | 20.51±0.22 |
| RCP 6.0/2080 | 21.57±1.02 | 17.8±0.26 | 20.16±0.46 | 25.11±0.84 | 22.07±0.46 | 19.54±0.52 | 18.08±0.30 | 18.26±0.57 | 20.7±0.56 | 19.56±0.38 |
| RCP 8.5/2020 | 23.48±0.34 | 19.59±0.25 | 22.02±0.22 | 27.99±0.30 | 19.79±0.19 | 20.91±0.24 | 19.71±0.33 | 20.07±0.35 | 22.48±0.17 | 21.03±0.20 |
| RCP 8.5/2050 | 21.11±0.44 | 18.72±0.27 | 19.49±0.44 | 25.66±0.70 | 21.89±0.49 | 19.29±0.50 | 18.07±0.30 | 18.6±0.59 | 21.09±0.37 | 19.84±0.27 |
| RCP 8.5/2080 | 19.37±0.68 | 16.47±0.37 | 18.58±0.71 | 22.93±0.98 | 20.28±0.74 | 17.71±0.73 | 16.69±0.48 | 16.68±0.80 | 18.88±0.37 | 18.26±0.50 |

**Supplementary Table S4. Variation in generation time of *Bactrocera correcta*** on mango in four scenarios under future climate change periods.

| Scenario/time period | Lucknow | Mohanpur | Paria | Ranchi | Rewa | Rupnagar | Bengaluru | Vengurle | Sangareddy | Dharampuri |
| --- | --- | --- | --- | --- | --- | --- | --- | --- | --- | --- |
| Baseline | 52.99 | 47.66 | 52.97 | 61.69 | 58.86 | 48.59 | 46.63 | 60.38 | 52.46 | 50.08 |
| RCP 2.6/2020 | 52.1±1.40 | 44.0±0.90 | 51.0±0.70 | 67.8±1.20 | 58.5±0.80 | 47.4±0.60 | 44.3±0.70 | 46.0±0.50 | 50.7±1.10 | 48.9±0.50 |
| RCP 2.6/2050 | 49.1±1.38 | 42.2±1.05 | 46.39±1.59 | 65.91±1.64 | 56.30±0.95 | 46.06±0.94 | 43.02±1.08 | 44.96±0.94 | 49.13±1.28 | 47.14±0.72 |
| RCP 2.6/2080 | 48.7±2.18 | 41.5±1.22 | 45.85±1.56 | 65.43±2.37 | 55.70±1.33 | 46.07±1.66 | 42.88±0.58 | 44.42±1.44 | 48.93±1.16 | 46.67±1.09 |
| RCP 4.5/2020 | 52.1±1.33 | 44.1±0.97 | 50.78±0.65 | 67.60±1.31 | 58.33±0.64 | 47.57±0.77 | 44.48±0.74 | 46.06±0.65 | 50.28±1.26 | 48.43±0.57 |
| RCP 4.5/2050 | 46.8±1.49 | 40.8±0.72 | 45.27±0.99 | 64.20±1.92 | 54.93±0.51 | 44.78±0.81 | 41.38±0.83 | 43.15±1.63 | 47.83±0.67 | 46.03±0.69 |
| RCP 4.5/2080 | 47.7±1.34 | 38.8±1.64 | 46.06±1.27 | 59.50±1.81 | 52.48±1.74 | 42.72±1.99 | 39.67±1.50 | 40.78±2.55 | 47.14±1.05 | 43.22±2.91 |
| RCP 6.0/2020 | 52.7±1.19 | 44.1±0.85 | 50.90±0.62 | 68.16±0.65 | 58.45±0.42 | 47.50±0.68 | 44.50±0.84 | 46.06±0.57 | 50.75±0.73 | 48.90±0.46 |
| RCP 6.0/2050 | 48.0±0.92 | 41.5±0.70 | 45.61±0.49 | 65.33±1.39 | 55.73±0.94 | 45.56±0.70 | 41.38±0.85 | 44.28±0.51 | 45.53±0.61 | 46.77±0.49 |
| RCP 6.0/2080 | 46.2±1.82 | 38.7±0.98 | 45.93±1.02 | 59.83±3.36 | 52.48±1.12 | 43.18±1.86 | 38.88±1.31 | 40.77±1.51 | 47.13±1.39 | 44.42±1.02 |
| RCP 8.5/2020 | 52.5±1.04 | 44.0±0.76 | 50.75±0.44 | 67.72±0.65 | 58.00±0.56 | 47.47±0.70 | 44.37±0.86 | 45.90±0.79 | 50.65±0.55 | 48.53±0.48 |
| RCP 8.5/2050 | 48.7±0.75 | 41.5±0.70 | 44.17±1.16 | 61.43±2.34 | 53.68±1.21 | 43.37±1.42 | 39.98±1.13 | 41.77±1.86 | 47.80±0.91 | 45.09±0.68 |
| RCP 8.5/2080 | 43.7±190 | 34.8±064 | 41.72±1.95 | 50.80±3.09 | 46.27±1.65 | 37.28±1.98 | 34.68±1.01 | 36.17±2.11 | 42.38±1.02 | 40.85±1.51 |
